# The good, the bad, and the unknown nature of decreased GD3 synthase expression

**DOI:** 10.3389/fnmol.2024.1465013

**Published:** 2024-11-22

**Authors:** Borna Puljko, Josip Grbavac, Vinka Potočki, Katarina Ilic, Barbara Viljetić, Svjetlana Kalanj-Bognar, Marija Heffer, Željko Debeljak, Senka Blažetić, Kristina Mlinac-Jerkovic

**Affiliations:** ^1^Croatian Institute for Brain Research, School of Medicine, University of Zagreb, Zagreb, Croatia; ^2^Department of Chemistry and Biochemistry, School of Medicine, University of Zagreb, Zagreb, Croatia; ^3^Department of Medical Chemistry, Biochemistry and Clinical Chemistry, Faculty of Medicine, Josip Juraj Strossmayer University of Osijek, Osijek, Croatia; ^4^Department of Neuroimaging, BRAIN Centre, Institute of Psychiatry Psychology and Neuroscience, King’s College London, London, United Kingdom; ^5^Department of Medical Biology and Genetics, Faculty of Medicine, Josip Juraj Strossmayer University of Osijek, Osijek, Croatia; ^6^Department of Pharmacology, Faculty of Medicine, Josip Juraj Strossmayer University of Osijek, Osijek, Croatia; ^7^Clinical Institute of Laboratory Diagnostics, Osijek University Hospital, Osijek, Croatia; ^8^Department of Biology, Josip Juraj Strossmayer University of Osijek, Osijek, Croatia

**Keywords:** ST8SIA1, gangliosides, glycosphingolipids, lipid metabolism, lipidomics

## Abstract

This paper explores the physiological consequences of decreased expression of GD3 synthase (GD3S), a biosynthetic enzyme that catalyzes the synthesis of b-series gangliosides. GD3S is a key factor in tumorigenesis, with overexpression enhancing tumor growth, proliferation, and metastasis in various cancers. Hence, inhibiting GD3S activity has potential therapeutic effects due to its role in malignancy-associated pathways across different cancer types. GD3S has also been investigated as a promising therapeutic target in treatment of various neurodegenerative disorders. Drugs targeting GD3 and GD3S have been extensively explored and underwent clinical trials, however decreased GD3S expression in mouse models, human subjects, and *in vitro* studies has demonstrated serious adverse effects. We highlight these negative consequences and show original mass spectrometry imaging (MSI) data indicating that inactivated GD3S can generally negatively affect energy metabolism, regulatory pathways, and mitigation of oxidative stress. The disturbance in several physiological systems induced by GD3S inhibition underscores the vital role of this enzyme in maintaining cellular homeostasis and should be taken into account when GD3S is considered as a therapeutic target.

## 1 Introduction

Gangliosides, complex glycosphingolipids, challenging to investigate with common molecular biology methods, are highly prevalent in the mammalian nervous system. Specifically, the human brain contains 10–30 times more gangliosides than any other organ (Svennerholm, 1994). Amongst those, GM1, GD1a, GD1b and GT1b represent 97% of all brain gangliosides (Yu et al., 2011). Gangliosides are synthesized by a series of specific glycosyl-and sialyltransferases in a metabolic pathway diverged into four branches (Yu et al., 2011). Hence, we can distinguish between asialo, a-, b-, and c-series of gangliosides according to the number of sialic acids (0, 1, 2, and 3) linked to the inner galactose residue (Schnaar, 2016, 2019). In this paper we are focusing on one of those sialyltransferases: GD3 synthase (GD3S) (alpha-*N*-acetylneuraminate alpha-2,8-sialyltransferase, EC 2.4. 99.8). GD3S regulates the biosynthesis of GD3 and GD2 from GM3, which are in turn starting points for the synthesis of more complex gangliosides of b-and c-series (Kasprowicz et al., 2022). It is encoded by the (*ST8SIA1, St8sia1*) gene (Kasprowicz et al., 2022) which is not solely expressed in the brain, but the RNA expression of *ST8SIA1* as well as expression of the protein product, GD3S, has been detected in a variety of other tissues, most prominently endocrine tissues, bone marrow, and lymphoid tissues, as illustrated in Supplementary Figure S1. Even though some tissues show a relatively modest RNA expression of the *ST8SIA1* gene, the protein expression is quite strong, e.g., liver and gallbladder, or the skin (Supplementary Figure S1). In this paper we aim to provide insight into the physiological consequences of decreased GD3S expression, accompanied by our own supportive observations and proposed future perspectives.

## 2 The good

Is achieving a favorable outcome realistic when considering the inhibition of any biosynthetic enzyme involved in ganglioside synthesis? Targeting this enzyme for inhibition has been a compelling research avenue, as elevated GD3S expression is associated with various pathological conditions, from cancer to neurodegenerative diseases.

GD3S has been recognized as one of the key factors in tumorigenesis (Tong et al., 2021). Overexpression of GD3S enhances tumor growth, proliferation, and metastasis in various cancers (Cazet et al., 2010; Schengrund, 2023) including thyroid, lung, colorectal, liver, pancreatic, renal, prostate, breast cancer, and most prominently, glioma and melanoma (Cazet et al., 2010; Yeh et al., 2016; Ma et al., 2017; Lee et al., 2018; Kasprowicz et al., 2022; Seo et al., 2024), illustrated in Supplementary Figure S2. Hence, inhibiting GD3S catalytic activity has raised interest as a potential therapeutic target considering its role in malignancy-associated pathways across different cancer types. Indeed, numerous studies have shown that decrease or abolishment of GD3S expression results in reduced malignancy, inhibition of proliferation, and decreased metastatic potential (Kasprowicz et al., 2022). Specifically, GD3S is highly expressed in GD2-positive breast cancer stem cells, and decreasing its expression results in inhibition of proliferation and self-renewal. This effect has also been demonstrated *in vivo* (Houghton et al., 1985). Promoting proliferation is induced by activating c-Met and the downstream targets MEK/ERK and the PI3K/AKT pathway (Ruan et al., 1999). GD3S was also found to promote metastasis of breast cancer by regulating epithelial-mesenchymal transition (Ruan et al., 1999). Furthermore, the absence of GD3S results in the downregulation of Akt, ERKs, and SFK phosphorylation in GD3S knock-out (GD3SKO) mouse glioma cells (Ohkawa et al., 2021).

Even though GD3S is responsible for the synthesis of b-and c-series gangliosides, ganglioside GD3 (Ohkawa et al., 2021) accounts for over 50% ganglioside content in glioblastoma and is most abundant in hyper-vascularized areas. It is mainly found in cell surface clusters and the cytoplasm of tumor cells (Hedberg et al., 2001). GD3-positive cells were detected in the peritumoral tissue up to 3.5 cm from the tumor edge, suggesting their implication in glioblastoma progression and invasion (Lama et al., 2016). In contrast, ganglioside GD3 is almost absent in the healthy adult human brain, making it a potential therapeutic target (Mennel et al., 2000; Iwasawa et al., 2018). Studies on GD3SKO mice found the animals develop tumors slower and the overall size of tumors was smaller than in wild-type (WT) mice, strengthening the notion that GD3S enhances the malignant properties of gliomas (Zhang et al., 2021). The introduction of GD3S cDNA into a neuroblastoma cell line (SH-SY5Y), which substantially expressed GM2 and GD1a but not GD3 and GD2, increased GD3 and GD2 expression, dispersed cell development and slowed down growth (Ruan et al., 1999).

GD3S has also been investigated as a potential novel therapeutic target in the context of various neurodegenerative disorders (Akkhawattanangkul et al., 2017). Huntington’s disease, Parkinson’s disease (PD), and Alzheimer’s disease (AD) are all characterized by altered ganglioside metabolism (Sipione et al., 2020). GD3S has been recognized as a novel AD treatment objective for cognitive decline, amyloid plaque development, and neurodegeneration (Bernardo et al., 2009). In a study on primary neurons and astrocytes lacking GD3S, Aβ-induced cell death and aggregation were prevented (Bernardo et al., 2009). Furthermore, APP/PSEN1/GD3S^−^/^−^ triple mutant mice experience improvements in behavioral deficits compared to APP/PSEN1 mice (Bernardo et al., 2009), hence elimination of GD3S improved memory and reduced amyloid-beta plaque loads (Bernardo et al., 2009). In the MPTP-induced (1-methyl-4-phenyl-1,2,3,6-tetrahydropyridine) PD model, suppression of GD3S exhibits neuroprotective qualities and may be a promising target for future research (Akkhawattanangkul et al., 2017). In another study, mice were intrastriatally injected with lentiviral-vector-mediated shRNA targeting GD3S (shGD3S) or scrambled-sequence control (scrRNA) and MPTP was administered. In shGD3S-treated mice, MPTP-induced lesions were smaller. MPTP caused bradykinesia and fine motor skill impairments in scrRNA-treated mice, while these deficiencies were absent in shGD3S-treated mice. These findings show GD3S inhibition prevents MPTP-induced nigrostriatal damage, bradykinesia, and fine motor skill impairments (Dhanushkodi et al., 2019). Furthermore, studies show that GD3 accumulation in plasma membrane lipid microdomains before mitochondrial translocation is crucial for Aβ-induced apoptosis (Kim et al., 2010). GD3S is rapidly activated in different cell types after apoptotic stimuli (Malisan et al., 2002), and GD3S overexpression was shown to cause vascular endothelial ECV304 cell death (Ha et al., 2004). Another research has provided evidence that drug-induced GD3S inhibition reduced CD95-induced apoptosis (De Maria et al., 1997). Additionally, GD3 causes mitochondrial membrane potential (ΔΨm) dissipation and swelling in isolated mitochondria, leading to cytochrome c, apoptosis-inducing factor, and caspase 9 release, prevented by enforced Bcl-2 activation (Rippo et al., 2000). In intact cells, suppression of GD3S expression significantly reduced ceramide-induced ΔΨm dissipation, demonstrating endogenous GD3 promotes mitochondrial alterations (Rippo et al., 2000).

The development of therapies targeted against GD3 and GD3S began several decades ago, utilizing various strategies, e.g., direct inhibition with monoclonal antibodies or stimulating the immune response with vaccines. Vaccine strategies have been developed mainly for potential treatment of malignant melanoma or other solid tumors (Yao et al., 1999). However, the clinical activity of some of them remained limited, leading to the cessation of vaccine development (Hein et al., 2024). Monoclonal antibodies, including R24, have been evaluated in clinical trials for melanoma patients. R24, a murine IgG3 anti-GD3 antibody, was tested in 61 patients with metastatic melanoma. Despite mild side effects, R24 confirmed the infiltration of immune cells in the tumor microenvironment following GD3 inhibition, opening new therapeutic developments (Houghton et al., 1985). Studies have shown GD3S inhibitors, such as Triptolide (TPL) from *Tripterygium wilfordii*, have anti-rheumatic, anti-inflammatory, immunomodulatory, and anti-tumor properties. TPL is a traditional Chinese medicinal herb that inhibits GD3S expression through NF-κB activation (Tong et al., 2021).

Furthermore, GD3SKO mice exhibit less age-related bone loss in skeletal tissue compared to WT mice (Yo et al., 2019). In GD3S KO mice, GD3 is not synthesized instead, other ganglioside species such as GD1a are present in higher amounts, and that increase could be linked to supporting bone maintenance and formation (Sasaki et al., 2022).

## 3 The bad

After establishing the positive effects of targeted GD3S inhibition, this section is to provide an overview of known negative consequences of decreased GD3S expression *in vitro*, available mouse models and human subject studies across different organ systems including the nervous system, retina, kidneys, and the liver.

GD3SKO mice, genetically engineered mice with nonfunctional *St8sia1* gene are an invaluable tool in the research of physiological and biochemical properties of GD3S and altered ganglioside composition on various cell functions. They are of normal growth and overall nervous tissue morphology, however, they exhibit thermal hyperalgesia and mechanical allodynia, decreased response to formalin-induced prolonged noxious stimulation (Handa et al., 2005), morphological abnormalities in the sciatic nerve, and neuronal disturbances during peripheral nerve regeneration (Ribeiro-Resende et al., 2014). In addition, they exhibit decreased hippocampal neuronal loss following global cerebral ischemia (Wang et al., 2021), impaired olfactory and memory functions due to reduced neurogenesis in the subventricular zone and the dentate gyrus (Fuchigami et al., 2024), impairment in hippocampus-dependent memory function (Tang et al., 2021), depression-like behavior (Wang et al., 2014), a reduction in rod and retinal ganglion cell populations and electrophysiological alterations in retinal ganglion cells, photoreceptors, bipolar and amacrine cells, and reduced contrast sensitivity and visual acuity (Abreu et al., 2021), as well as mild impairment of spontaneous regeneration of neuromuscular junctions in older animals (Rupp et al., 2013). Analysis of neuronal stem cells (NSCs) obtained from GD3SKO mice has shown decreased self-renewal ability compared with those from the WT animals, accompanied by reduced expression and increased degradation rate of EGF receptor (Wang and Yu, 2013).

Ganglioside metabolism has been thoroughly examined in the neuronal retina (Panzetta and Allende, 2000), with substantial GD3S activity shown in the early and late development of rat and chicken retinas (Daniotti et al., 1991, 1992, 1994; Watanabe et al., 1996; Maxzúd and Maccioni, 1997; Panzetta and Allende, 2000). *St8sia1* has been identified as a potential candidate in the acquisition of experience-dependent plasticity in murine visual cortex (Rietman et al., 2012), and polymorphisms in the human *ST8SIA1* have been identified in patients with treatment-resistant ophthalmoplegia (Nel et al., 2017). The visual system of GD3SKO mice exhibits a reduction in retinal ganglion cell (RGC) density, optic nerve fiber number, RGC and photoreceptor electrical activity, visual acuity, and contrast sensitivity, indicating b-series gangliosides are essential for visual system structure and function (Abreu et al., 2021). Furthermore, gangliosides synthesized by GD3S, are present in the inner ear and contribute to the maintenance of the structure and function of auditory cells (Yoshikawa et al., 2009, 2015; Inokuchi et al., 2017; Kurabi et al., 2017).

Other organs are also affected by the lack of GD3S. A study on GD3SKO mice has shown changes in the expression of renal connexins and pannexin1 (Meter et al., 2022). Ganglioside GM3, accumulated in GD3SKO mice, has been documented in podocytes (Savas et al., 2020) and was related to glomerular hypertrophy occurring in diabetic human and rat kidneys (Novak et al., 2013). Furthermore, advanced glycation end products were shown to inhibit bovine retinal pericyte and rat renal mesangial cell proliferation associated with GD3S activity inhibition suggesting its role in the development of diabetic retinopathy and diabetic nephropathy (Masson et al., 2005). Human pancreatic islets of Langerhans contain five distinct endocrine *β*-4 cell types, which are distinguished by differential expression of *ST8SIA1* and *CD9,* and their distribution is altered in type 2 diabetes (Dorrell et al., 2016). Gene expression analysis suggests *ST8SIA1^−^* β cells secrete more insulin (Dorrell et al., 2016). Correlation analysis has revealed *ST8SIA1* as one of the genes with integrative changes in RNA expression and DNA methylation in the offspring born to women with pregestational type 1 diabetes (T1DM) (Knorr et al., 2022). Furthermore, in GD3SKO mice liver, a prominent difference in expression of cholesterogenic genes *Srebp1a*, *Insig2a*, and *Ldl* was found, indicating a relationship between gangliosides and regulation of cholesterol metabolism (Mlinac et al., 2012). With everything stated, keeping in mind the high RNA and protein expression of GD3S in multiple organs and tissues (Supplementary Figure S1), the consequences of GD3S inhibition are under-investigated and need to be explored further.

## 4 The unknown

The complete spectrum of proteins and lipids interacting with GD3S and the functional consequences of these interactions are not well-documented. Identifying them could provide insight into broader cellular processes influenced by GD3S. Additionally, how GD3S activity integrates with other metabolic and signaling pathways under different physiological conditions is still unknown.

To explore the relationship between gangliosides and metabolic pathways, we analyzed nucleus caudatus and putamen (together known as the corpus striatum (CPu), an important part of the basal ganglia) of 4.5 months old GD3SKO and WT mice using mass spectrometry imaging (MSI). The obtained spectra were used for further data processing to observe differences in the lipidome between GD3KO and WT mice. Compared to WTs, CPu region in GD3SKO mice shows significantly reduced (negative t-scores greater than 2 in absolute value) expression of 8 compounds functionally involved in energy metabolism (Table 1). Putatively identified compounds indicate additional not yet identified effects of GD3S and suggest inhibition of GD3S may affect pathways linked to energy production (Table 1).

**Table 1:** Selected putatively identified significant m/z signals and *t*-scores detected by mass spectrometry imaging (MSI)* in the nucleus caudatus and putamen (CPu) region that are reduced in GD3SKO mice compared to WT mice.

KEGG ID	m/z	Name of identified compaund	KO vs WT *p* values	*t*-score	Pathway
C04145	1580.02	All-trans-Nonaprenyl diphosphate	0.03	−3.70880	Ubiquinone biosynthesis
C00894	1641.26	Propenoyl-CoA, Acryloyl-CoA	0.03	−4.06603	Propanoate metabolism
C05989	1673.22	3-Oxopropionyl-CoA, Malonyl semialdehyde-CoA	0.03	−3.98330	Propanoate metabolism
C15670	1703.7	Heme A	0.04	−3.33664	Porphyrin metabolism
C11378	1724.36	Ubiquinone-10, Coenzyme Q10	0.04	−3.43541	Ferroptosis
C05268	1761.34	(S)-Hydroxyhexanoyl-CoA	0.04	−3.51413	Fatty acid elongation and degradation
C05265	1869.46	3-Oxodecanoyl-CoA	0.04	−3.34195	Fatty acid elongation and degradation
C00406	1885.3	Feruloyl-CoA, 4-Hydroxy-3-methoxycinnamoyl-CoA	0.04	−2.94010	Arginine and proline metabolism

KO – GD3SKO mice, WT – wild-type mice, *p* < 0.05. *Imaging mass spectrometry was performed using Shimadzu IMScope TRIO MALDI-IT-TOF (Shimadzu, Kyoto, Japan). Tissue sections were mounted on ITO slides. The matrix (9-aminoacridine) was applied to samples using iMLayer device (Shimadzu, Kyoto, Japan) which included a 15 min sublimation at 180°C, followed by 5 min recrystallization at 37°C with 5% methanol in a vapor chamber. Imaging was performed in negative ion mode within an m/z range of 1,500–1900 Dalton and the following setup: laser diameter 25 μm, laser intensity 46%, 100 laser shots/pixel. Pixel number was 3,000. Data analysis was performed in R software ver. 4.0.1. The expression of a compound is reduced in the CPu region of KO mice when its *t*-score value is negative.

Putatively identified compounds include propenoyl-CoA and 3-oxopropionyl-CoA which are part of the propanoate metabolism encompassing the metabolism of propionic acid. Propionyl-CoA is a key intermediate in the breakdown of odd-numbered fatty acids as well as some amino acids. Aberrations in this pathway are associated with various diseases, such as primary propionic acidemia and malonyl-CoA decarboxylase deficiency (Pena et al., 2012). In addition, metabolites from fatty acid metabolism can impact other pathways, including lipid and glycosphingolipid synthesis and are decreased in GD3SKO mice (Table 1). Peroxisome proliferator-activated receptors (PPARs), especially PPARα, regulate fatty acid metabolism and influence the expression of lipid metabolism genes (Hu et al., 2023), affecting ganglioside biosynthesis.

Moreover, decreased expression of ubiquinone 10 (coenzyme Q10), a component of the mitochondrial electron transport chain, is related to a range of neurologic, renal, cardiac, and other clinical manifestations (Hargreaves et al., 2020; Mantle et al., 2023). Decreased Q10 synthetic pathway is supported by decreased expression of all-trans-nonaprenyl diphosphate which is important for biosynthesis of ubiquinone and other terpenoid-quinones. Ubiquinone acts as an antioxidant, protecting cells from oxidative damage and reducing stress, vital for maintaining enzyme activities and cellular signaling (Gutierrez-Mariscal et al., 2020). Similarly, heme, which is also identified in our study (Table 1) helps maintain cellular redox balance through the electron transport chain, influencing enzyme expression and function. Heme A is a crucial component of molecular systems required for oxygen transport and cellular respiration. Based on reduced expression of ubiquinone and heme A, GD3S could be related to increased oxidative stress in the cells. In addition, enhanced sulfur metabolism affects the redox state and overall metabolic profile, potentially modulating GD3S-related pathways and influencing ganglioside synthesis involved in tumor growth and metastasis.

When analyzing the consequences of GD3S downregulation or inactivation, other research not directly investigating the mouse or cellular GD3SKO models needs to be considered. It is established that decreased GD3 levels might compromise lipid raft integrity and function, altering cellular communication and signal transduction (Sonnino and Prinetti, 2010). Because the integrity and functionality of cellular membranes are also essential for the correct operation of ion channels and transporters, a decrease in GD3S activity can seriously disturb ion homeostasis. This may result in extensive cellular malfunction, affecting functions like potassium and sodium homeostasis and calcium signaling (Bretscher and Munro, 1993; Ledeen et al., 1998; Simons and Gerl, 2010). For example, in a recent paper exploring ganglioside interactome (Zhang et al., 2024), specific ganglioside-protein interactions were identified, previously anticipated by literature (Ilic et al., 2021; Puljko et al., 2022). They include particular subunits of Na^+^/K^+^ ATPase and plasma membrane calcium ATPase (Zhang et al., 2024). Since specific gangliosides directly bind these proteins, changed ganglioside composition caused by GD3S inhibition and the consequent lack or accumulation of specific ganglioside species could lead to their disturbed activity and function. In addition, the reshaped ganglioside milieu leads to altered membrane fluidity and consequently dysfunctional ion channels, impacting the cell’s ability to maintain proper ion gradients (Puljko et al., 2021) which adds an additional energy-demanding challenge. Furthermore, decreased activity of GD3S has the potential to interfere with calcium signaling pathways and disturb intracellular calcium homeostasis (Ledeen et al., 1998), which has severe implications across multiple signaling pathways since Ca^2+^ is a second messenger. Also, gangliosides play a role in modulating cell–cell/cell-matrix interactions, which are critical for maintaining tissue structure and function, and lower activity of GD3S can impair cell adhesion and migration, potentially affecting tissue integrity and repair mechanisms (Hakomori Si, 2002).

## 5 Conclusions and perspectives

**Figure 1:**
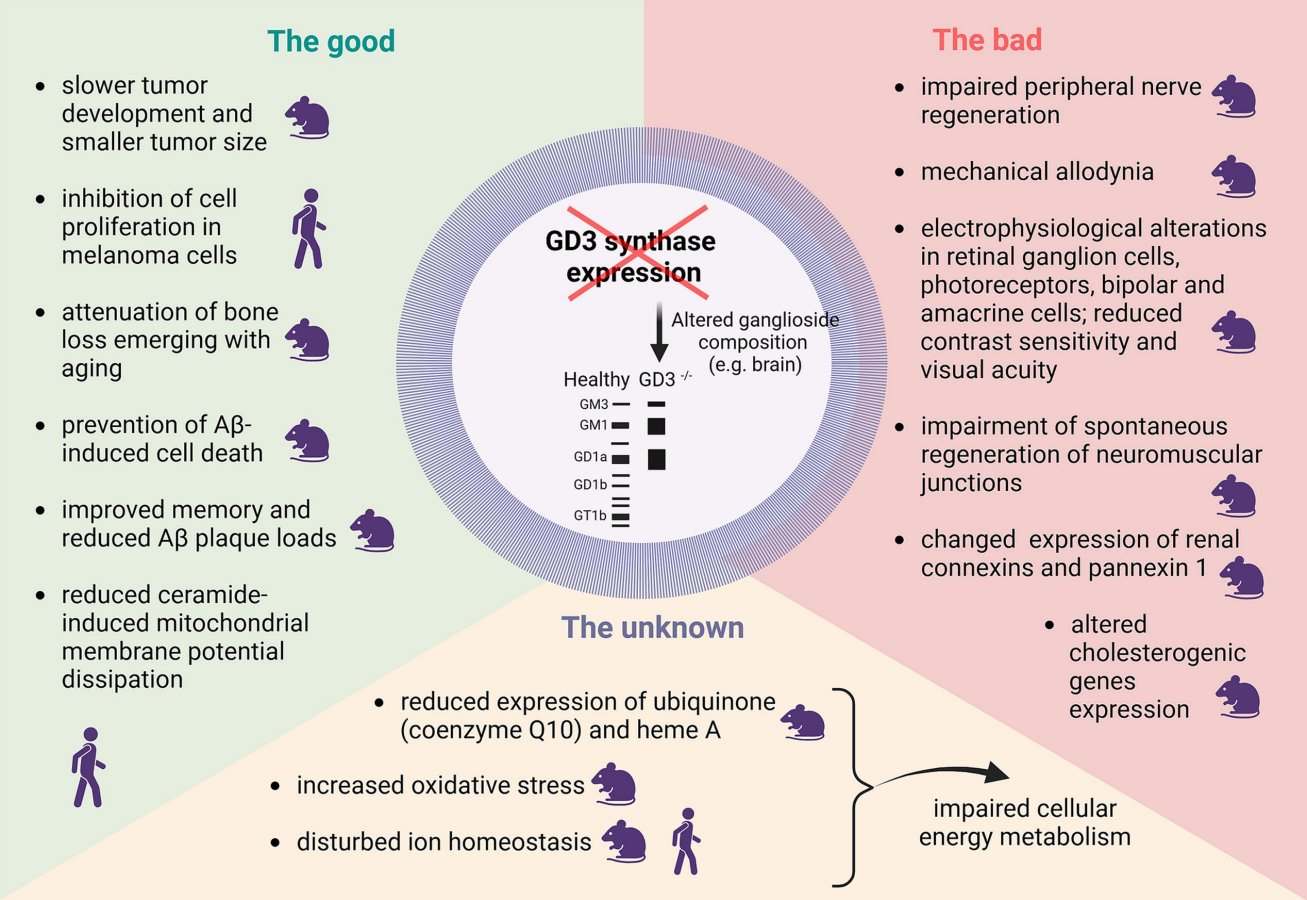
Schematic representation of the good, bad and the unknown consequences of decreased GD3 synthase expression. The human or mouse icon highlight whether the research was performed in mouse or human cell lines and/or tissue/ animal models. The figure was created with Biorender.com.

The known, both good and bad, as well as hypothesized consequences of decreased GD3S expression, are schematically shown in Figure 1. Altered GD3S activity and consequent changed b-series gangliosides synthesis are important contributors to various pathologies, including tumors and neurodegenerative diseases. GD3S overexpression is linked to enhanced tumor proliferation and metastasis in multiple cancers while inhibition of GD3S is a potential therapeutic strategy due to its role in malignancy-associated pathways, evidenced by studies showing reduced malignancy and metastasis upon GD3S suppression (Figure 1). On the other hand, decreased activity of GD3 synthase has significant implications for disturbed cellular homeostasis, including impairing immune responses, cellular adhesion, signal transmission, and membrane integrity. Our own MSI data (Table 1) shows decreased expression of the putatively identified compounds as the consequence of GD3S inactivation in GD3SKO mice which can generally negatively affect energy metabolism, regulatory pathways, and mitigation of oxidative stress.

The possible effects of the disturbances on several physiological systems underscore the vital role of GD3S in cellular homeostasis. Further studies should try to elucidate the intricate regulatory pathways governing the expression and activity of GD3S, analyze its diverse functional roles across different tissues, and explore its potential as a therapeutic target in pathological conditions such as cancer, neurodegenerative diseases, and immune disorders. Understanding the molecular underpinnings of GD3S may facilitate the creation of novel strategies for the detection, management, and prevention of a variety of disorders, creating new avenues for targeted therapies and exploiting the role of GD3 synthase in cellular processes for clinical benefit.

## Data Availability

The original contributions presented in the study are included in the article/Supplementary material, further inquiries can be directed to the corresponding authors.
