# Supplementary material for: The good, the bad, and the unknown nature of decreased GD3 synthase expression
Source: Front Mol Neurosci. 2024 Nov 22;17:1465013. doi: 10.3389/fnmol.2024.1465013 (PMC11621222; doi:10.3389/fnmol.2024.1465013)
Supplement: Supplementary file 1 [file Data_Sheet_1.PDF]

Supplementary Material

The good, the bad, and the unknown nature of decreased GD3 synthase expression

Borna Puljko[1,2], Josip Grbavac[3], Vinka Potočki[1], Katarina Ilic[4], Barbara Viljetić[3], Svjetlana Kalanj-Bognar[1,2], Marija Heffer[5], Željko Debeljak[6,7], Senka Blažetić[8*], Kristina Mlinac-Jerkovic[1,2*]

[1] Croatian Institute for Brain Research, School of Medicine, University of Zagreb, Zagreb, Croatia

[2] Department of Chemistry and Biochemistry, School of Medicine, University of Zagreb, Zagreb, Croatia

[3] Department of Medical Chemistry, Biochemistry and Clinical Chemistry, Faculty of Medicine, Josip Juraj Strossmayer University of Osijek, Osijek, Croatia

[4] BRAIN Centre, Department of Neuroimaging, Institute of Psychiatry, Psychology and Neuroscience, King's College London, UK

[5] Department of Medical Biology and Genetics, Faculty of Medicine, Josip Juraj Strossmayer University of Osijek, Osijek, Croatia

[6] Department of Pharmacology, Faculty of Medicine, Josip Juraj Strossmayer University of Osijek, Osijek, Croatia

[7] Clinical Institute of Laboratory Diagnostics, Osijek University Hospital, Osijek, Croatia

[8] Department of Biology, Josip Juraj Strossmayer University of Osijek, Osijek, Croatia

*** Correspondence:**
Corresponding Authors: Senka Blažetić*, Kristina Mlinac-Jerkovic*
senka@biologija.unios.hr
kristina.mlinac.jerkovic@mef.hr

1.1 Supplementary Figures

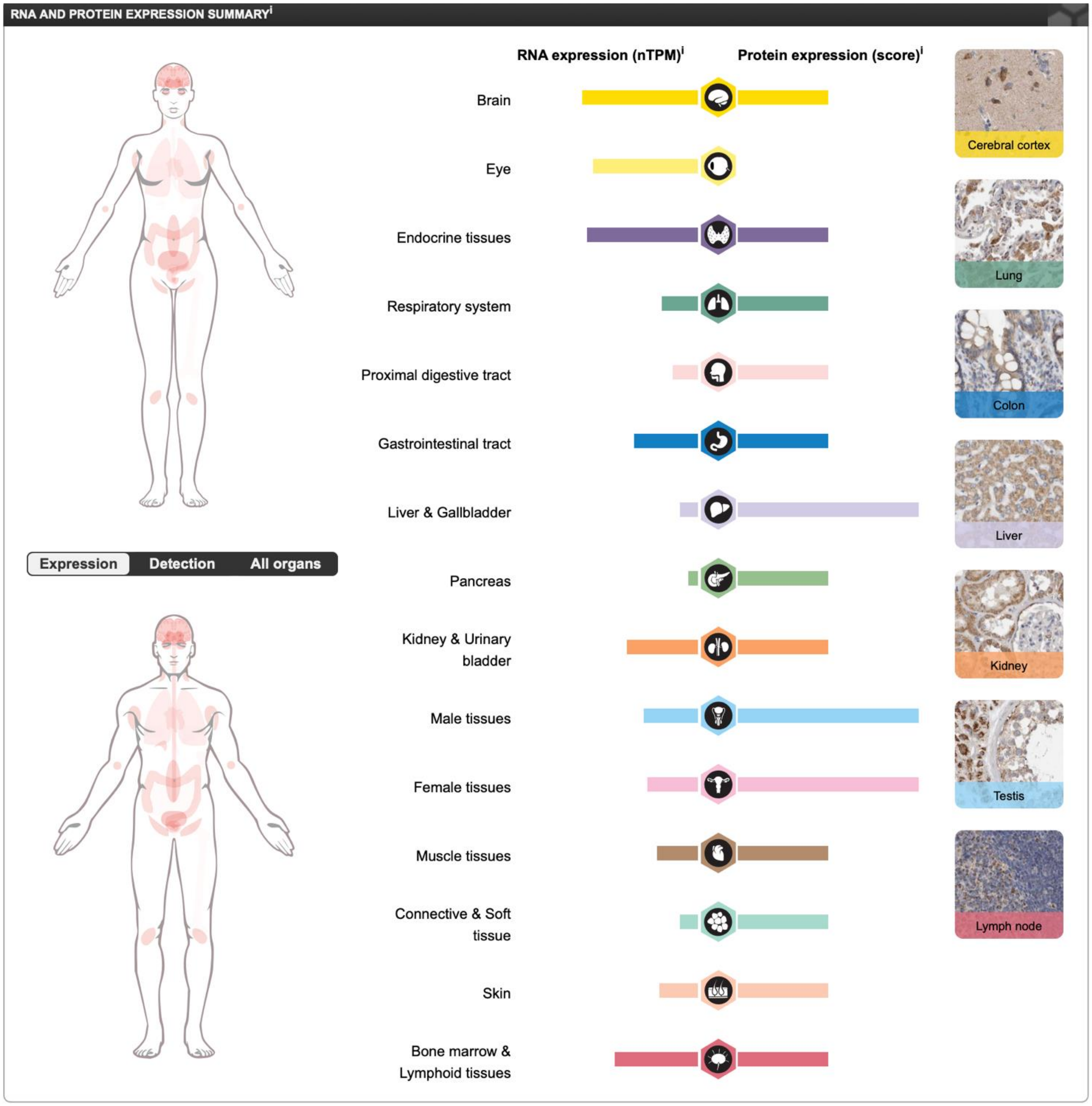

Supplementary Figure 1. Overview of *ST8SIA1* RNA expression data as determined by the Human Protein Atlas project. Analyzed tissues are divided into color-coded groups according to which functional features they have in common. The IHC image panel to the right displays selected tissues that give a visual summary of the protein expression profile. To the left, two human bodies provide an anatomical display of the expression levels and detection of *ST8SIA1* mRNA in the analyzed organs (1). RNA expression summary shows the consensus data based on normalized expression (nTPM) values from two different sources: internally generated Human Protein Atlas (HPA) RNA-seq data and RNA-seq data from the Genotype-Tissue Expression (GTEx) project. Color-coding is based on tissue groups, each consisting of tissues with functional features in common. Each bar represents the highest

expression score found in a particular group of tissues. Protein expression scores are based on a best estimate of the "true" protein expression from a knowledge-based annotation, described more in detail under Assays & annotation. For genes where more than one antibody has been used, a collective score is set displaying the estimated true protein expression. Image credit: Human Protein Atlas.

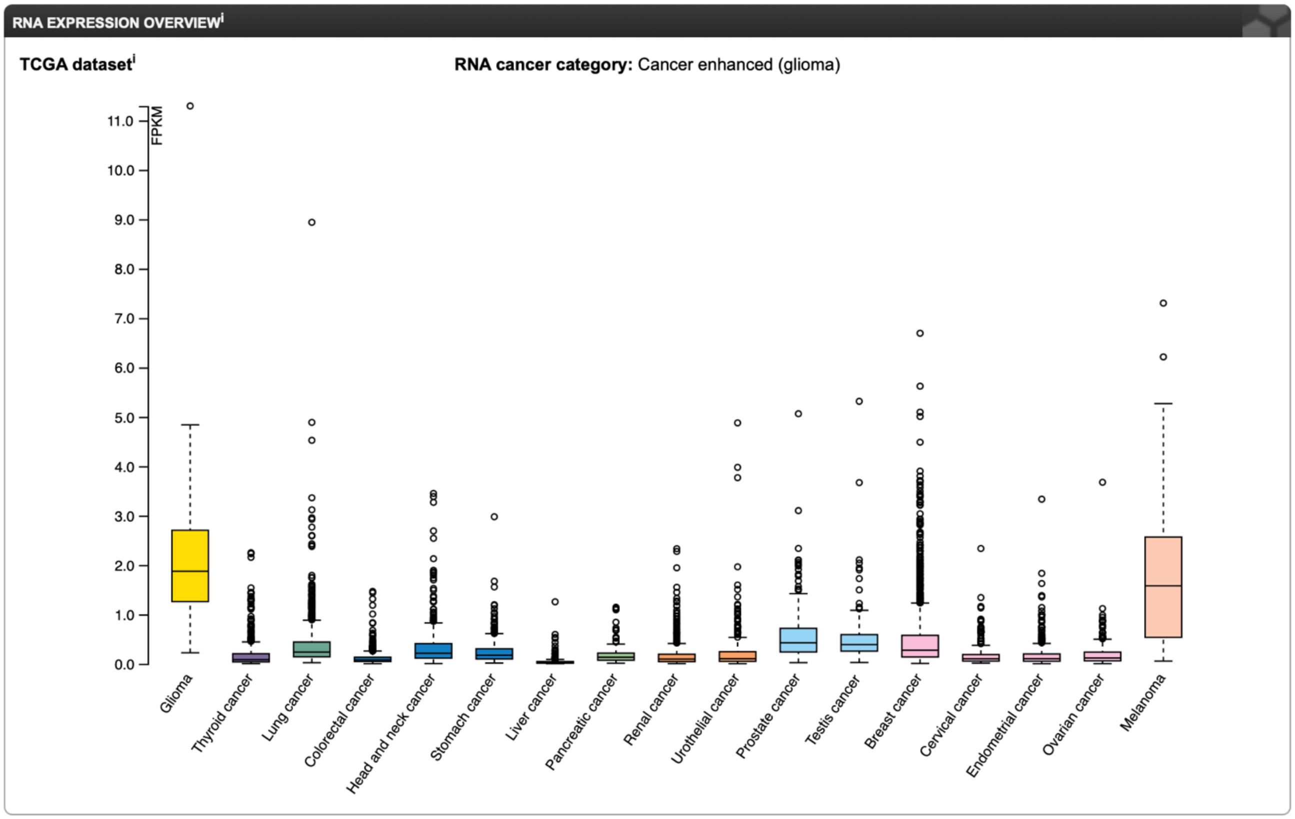

Supplementary Figure 2. ST8SIA1 RNA expression showing RNA-seq data from The Cancer Genome Atlas (TCGA) (2). Normal distribution across the dataset is visualized with box plots, shown as median and 25th and 75th percentiles. Points are displayed as outliers if they are above or below 1.5 times the interquartile range. To access cancer specific RNA and prognostic data, click on the cancer name. The cancer types are color-coded according to which type of normal organ the cancer originates from. Image credit: Human Protein Atlas.

Supplementary References:

1. Tissue expression of ST8SIA1 - Summary - The Human Protein Atlas [Internet]. [cited 2024 Jun 29]. Available from: https://www.proteinatlas.org/ENSG00000111728-ST8SIA1/tissue

2. Expression of ST8SIA1 in cancer - Summary - The Human Protein Atlas [Internet]. [cited 2024 Jun 29]. Available from: https://www.proteinatlas.org/ENSG00000111728-ST8SIA1/pathology
